# Health behaviors of people with multiple sclerosis and its associations with MS related outcomes: a German clinical cohort

**DOI:** 10.3389/fneur.2023.1172419

**Published:** 2023-09-13

**Authors:** Katharina Goldin, Karin Riemann-Lorenz, Anne Daubmann, Jana Pöttgen, Nicole Krause, Helmut Schröder, Christoph Heesen

**Affiliations:** ^1^Institute of Neuroimmunology and Multiple Sclerosis, University Medical Center Hamburg-Eppendorf, Hamburg, Germany; ^2^Institute of Medical Biometry and Epidemiology, University Medical Center Hamburg-Eppendorf, Hamburg, Germany; ^3^IMIM-Hospital del Mar Medical Research Institute, Barcelona, Spain; ^4^CIBER Epidemiologia y Salud Pública (CIBERESP), Madrid, Spain

**Keywords:** multiple sclerosis, health behaviors, diet, physical activity, tobacco smoking, MS related health outcomes, clinical cohort

## Abstract

**Background:**

Health behaviors in persons with multiple sclerosis (pwMS) have been associated with MS-related disease outcomes.

**Objective:**

The aim of the study was to gain knowledge about current patient health behaviors in a convenience sample representative for pwMS presenting to a large university-based outpatient clinic and to investigate associations between modifiable risk factors with physical impairment, quality of life (QoL) and cardiovascular comorbidities.

**Methods:**

A questionnaire was administered at the MS Outpatient Clinic of the University Medical Center Hamburg Eppendorf asking for health behaviors regarding dietary habits assessed with the German adaptation of the validated Spanish short Diet Quality Screener (sDQS), level of physical activity assessed with the Godin Leisure Time Questionnaire (GLTEQ) and tobacco smoking. Participants were asked to report cardiovascular comorbidities using items from the Self-Report Comorbidity Questionnaire for Multiple Sclerosis. Additionally, cardiovascular risk factors like blood pressure, height and weight (to calculate BMI) and waist circumference were measured. MS specific clinical data, e.g., disease course, duration, disability and MS-specific QoL were collected from the clinical database. Descriptive analyses were performed and multivariate regression analyses for complete cases were carried out for each of the three outcome variables including all mentioned modifiable risk factors (dietary behavior, smoking, physical activity and BMI) as independent variables.

**Results:**

In this sample of 399 pwMS the mean age was 42 years (SD 12.8) with a mean disease duration since diagnosis of 7.4 years (SD 8.4) and a mean EDSS of 2.8 (SD 1.9). 24% were current smokers, 44% were insufficiently physically active and 54% did not follow a healthy dietary pattern. 49% of this relatively young clinical population was overweight and 27% reported one or more cardiovascular comorbidities. Most modifiable risk factors showed no convincing associations with MS-related disease outcomes in the multiple regression analyses.

**Conclusion:**

This clinical cohort of pwMS shows a high prevalence of critical health behaviors and comorbidities and emphasizes the need for monitoring, education and assistance for behavior change in this population.

## 1. Introduction

Multiple sclerosis (MS) is an inflammatory and degenerative disease of the central nervous system (CNS). The worldwide incidence increased over the last decades (1). In Germany approximately 240,000 people are living with MS (2). Many experience impairments in vision, mobility, cognitive function and bladder control (3) and suffer from symptoms like pain, spasticity, fatigue and comorbid depression (4), which substantially impact on quality of life in persons with multiple sclerosis (pwMS).

The risk of being affected with MS is multifactorial and can partly be explained by genetic predisposition coupled with a variety of health behaviors and environmental factors, e.g., latitude and corresponding low Vitamin D levels (5). Additionally, a historical Epstein–Barr virus infection enhances the risk of developing MS, indicating a pathogenetic role of viral antigen immune system interaction (6). Environmental exposures and modifiable risk factors, such as diet, smoking, physical activity and obesity have been linked to epigenetic changes that may contribute to MS development and progression (7).

As one modifiable risk factor, dietary habits have been associated with progression and symptom severity in pwMS. A lower dietary quality such as a high intake of saturated fats, processed foods, red meat or low intake of fruits and vegetables, has been associated with a higher disability level and more severe depression symptoms (8–11). Cross-sectional studies using data from the Australian MS Longitudinal Study (AMSLS) (12) and from the UK Multiple Sclerosis Registry (9), found that high fiber and fruit/vegetable consumption was associated with better health outcomes, especially a better health-related QoL in pwMS (9, 11, 12). Moreover, in a large cross-sectional study with patients of the North American Research Committee on MS (NARCOMS) registry, a healthy diet was associated with reduced symptom severity and disability (8). Immune modulation, neuroprotection and nervous system repair may be the explanatory mechanisms that can be achieved through a healthy diet (10). There is also emerging evidence that the gastrointestinal microbiota may play a role in mediating the influence of diet on MS pathogenesis (13). Due to the heterogenous research field in terms of content and methodology concerning diets, nutrients and supplements, high quality studies are still lacking. As there is an increasing interest among pwMS to modulate their disease progression through behavior change, randomized controlled trials (RCTs) need to be conducted to be able to give pwMS evidence based recommendations.

Cigarette smoke contains numerous harmful components, such as free radicals and toxins, which can directly damage the cells of the CNS and exacerbate oxidative stress (14). Smoking increases the risk of developing MS (15) and is associated with a lower QoL and higher impairment (16). Although smoking cessation has significant benefits for pwMS (17), no programs have been evaluated in MS populations.

Physical activity including (high intensity) exercise is known to be another potential modifiable factor influencing MS progression. Research has shown that physical activity is associated with improved clinical outcomes in pwMS such as higher QoL and reduced impairment (18) and therefore physical activity guidelines for pwMS are available (19). Exercise may modulate anti-inflammatory effects and could enhance immune regulation, but detailed pathophysiology remains uncertain due to the complexity of influences (20).

Another modifiable risk factor that has gained considerable attention is obesity and its associated metabolic dysregulation. A recent study has demonstrated an increased risk of developing MS and a more severe disease course in obese individuals (21). Adipose tissue is known to produce pro-inflammatory cytokines and adipokines, which can contribute to chronic systemic inflammation (22). Research on detailed pathophysiology of the link between obesity and MS is in progress.

Moreover, there is evidence from registry studies that comorbidities impact negatively on disease progression, cognition, mortality, and health-related QoL (23–25). Especially vascular comorbidities like hypertension, diabetes, heart disease and hypercholesterolemia were associated with a substantially increased risk of disability progression in a large sample of the NARCOMS registry (26) and in a Belgrade MS population Registry cohort (23). Health behaviors such as physical activity, dietary pattern, tobacco smoking and Body Mass Index (BMI) are known to be associated with cardiovascular risk factors and vascular comorbidities (25).

These numerous modifiable risk factors might also partly be responsible for the highly variable disease activity and progression in MS (27, 28). Although Disease Modifying Treatments (DMTs) are able to reduce inflammation and the accumulation of lesions on Magnetic Resonance Imaging, the effect on disability progression is only moderate (29). Hence, rehabilitation and secondary prevention remain important strategies for managing symptoms and dysfunction (30, 31).

Despite these observations there is evidence from international studies that the prevalence of suboptimal health behaviors and overweight/obesity is high among pwMS (12, 32, 33). To date, little is known about health behaviors of pwMS in Germany. The aim of the present study was to describe health behaviors of a German clinical cohort of pwMS in a large university-based outpatient clinic and to explore if diet, tobacco smoking, physical activity and BMI are associated with physical impairment, quality of life and cardiovascular comorbidities.

## 2. Materials and methods

### 2.1. Participants and recruitment

Data were collected from a consecutive convenience sample of *n* = 427 pwMS treated at the MS Outpatient Clinic of the University Medical Center Hamburg Eppendorf (UKE) from August 2020 to June 2021. For logistical reasons, only patients who had an appointment at the MS Outpatient Clinic on single days of each week could be recruited. Participants were eligible for the study if they had a definite MS diagnosis according to the 2017 McDonald criteria (34). After excluding 28 participants who did not meet these criteria, *n* = 399 patients with a confirmed MS diagnosis were included in the study. In order to evaluate the representativeness of the sample we used demographic data (age, sex and school education) and level of impairment [MS-specific Expanded Disability Status Scale (EDSS)] of all pwMS who presented to the MS Outpatient Clinic from August 2020 to June 2021 (*n* = 894) to compare the study population (*n* = 399) with the patient group not included in the study (*n* = 495). The questionnaire was administered to patients, who got either a control examination at the Outpatient Clinic or visited it for initial presentation. In both cases, MS specific measurements such as EDSS, Hamburg Quality of Life Questionnaire in Multiple Sclerosis (HAQUAMS), 9-Hole Peg Test (9-HPT) and Timed 25-Foot Walk Test (T25-FW) were always assessed in parallel to filling-out the questionnaire. Demographic data as well as the date of onset of MS to calculate the disease duration were used from the clinical database. Information about school education is routinely queried at the initial presentation at the Outpatient Clinic and categorized as school attendance of less or more than 12 years. This study was carried out as part of the Hamburg MS registry project evaluated by the Ethics Committee of the Hamburg Chamber of Physicians. Informed consent was signed by each participating patient.

### 2.2. Measurements and scales

Data for this study were obtained from three sources: Clinical measurements, self-report questionnaires and the clinical database of the MS Outpatient Clinic.

#### 2.2.1. Clinical measurements

##### 2.2.1.1. Blood pressure

Blood pressure was assessed according to the ESC/ESH guidelines (35) using digital, automated upper arm monitors. If the systolic blood pressure was above 140 mmHg and/or diastolic blood pressure above 90 mmHg, the measurement was repeated after the patient rested for 5 min.

##### 2.2.1.2. Body Mass Index

Participants were weighed by clinical staff (without shoes and with light clothing) and their height was measured. The BMI is defined as a person’s weight in kilograms divided by their squared height in meters (kg/m^2^). Evaluation was based on World Health Organization (WHO) criteria indicating that a BMI below 18.5 means underweight, 18.5–24.9 normal weight, 25–29.9 pre-obesity and above 30 obesity (36).

##### 2.2.1.3. Waist circumference

The waist circumference was measured by clinical staff in a standing position at the midpoint between the lower rib and the top of the hip bone and was assessed according to the WHO criteria (37). Two sex-specific cut-off points in the Caucasian population which indicate increased risk for metabolic complications were applied: (a) males with a waist circumference of ≥94 cm and females ≥80 cm and (b) males ≥102 cm and females ≥88 cm have a substantially increased risk. The waist circumference was rated as “high” when the first cut-off point was exceeded (37).

#### 2.2.2. Questionnaire

##### 2.2.2.1. Short diet quality screener

Dietary assessment was performed with a German adaptation of the validated Spanish short Diet Quality Screener (sDQS) which has been developed in cardiovascular epidemiology (38, 39). While forward and backward translations had been performed before implementation in the actual study, a formal investigation on the construct validity is ongoing. The screener aims to quickly estimate the quality of the dietary pattern. Study participants were asked to report their average consumption of 17 food groups over the last 4 weeks. Daily frequency consumption was assessed for the following food groups: whole grain bread, vegetables, fruits, milk/dairy products and cheese, other whole grain products, plant oil and calorie-free drinks. Weekly consumption frequency was assessed for meat, meat products, pastries/cake, sweets, butter/lard/coconut fat, sweetened beverages, fast food, fish, legumes and nuts (for details see Appendix A1). To calculate the sDQS score all food item scores were added up. The total possible score ranges from 17 to 51. In close consultation with the developer of the sDQS, we calculated tertiles for descriptive purposes: Scores of 40–51 are rated as a “healthy diet,” scores between 28 and 39 as a “moderately healthy diet” and scores between 17 and 27 are rated as an “insufficiently healthy diet.” Continuous scores of the sDQS were used for the regression analyses.

##### 2.2.2.2. Godin leisure time exercise questionnaire

The Godin Leisure Time Exercise Questionnaire (GLTEQ) is a brief and validated questionnaire asking about the frequency of usual leisure-time exercise for more than 15 min (strenuous, moderate, mild) in a typical 7-day week (40). Strenuous activities like jogging correspond to nine Metabolic Equivalents of Task (METs), moderate activities like tennis correspond to five METs and mild activities like yoga correspond to three METs. The total score is computed by summing up the products and ranges from 0 to 119 (40). Based on patient-reported frequency of moderate-to-vigorous physical activity, the Health Contribution Score (HCS) from the GLTEQ was assessed ranging from 0 to 98 (41). According to Godin (41) participants who score 24 units and more are rated as “active” with substantial benefits for health. Persons scoring 14–23 units are rated as “moderately active” with some health benefits and participants who score below 14 units are rated as “insufficiently active” with less or low health benefits (41).

##### 2.2.2.3. Tobacco smoking behavior

Smoking behavior was assessed based on self-report and categorized into “never-smokers” (defined within the questionnaire as those who smoked rarely or have smoked less than 100 cigarettes in their lives), “current smokers” and “ex-smokers.” Current smokers and ex-smokers were asked about their smoking habits in greater detail, but these data have not been used for the current analysis. Questions on smoking behavior were based on common recommendations on smoking behavior survey in epidemiological studies (42).

##### 2.2.2.4. Cardiovascular comorbidities

Cardiovascular comorbidities were assessed based on the Self-Report Comorbidity Questionnaire for Multiple Sclerosis by Horton et al. (43) The validation of this questionnaire in a Canadian population showed that agreement between self-report and medical records was high for diabetes and hypertension, substantial for hyperlipidemia and moderate for heart disease. The authors concluded that self-report was a reasonable way to capture the presence of comorbidities among pwMS (43). Participants were asked if they were diagnosed with one or more of six cardiovascular comorbidities: dyslipidemia, high blood pressure, diabetes type 1 or 2, peripheral vascular disease or a heart disease. The questions included both, lay person terminology and clinical terms. If study participants marked none of them, it was rated as “no comorbidities.”

#### 2.2.3. MS clinical database variables

##### 2.2.3.1. Disease course

One of the following disease courses were documented for all participants in the clinical database: Relapsing-remitting MS (RRMS), primary progressive MS (PPMS), secondary progressive MS (SPMS), “MS type not yet defined” or “other.”

##### 2.2.3.2. Expanded Disability Status Scale

The EDSS quantifies MS disability based on neurological examination performed by neurologists and is a valid measure in clinical practice and research. Reliability remains under critic, because interpretation depends on the examiner. The scale ranges from 0 to 10 and increases in 0.5 steps with higher scores representing higher levels of impairment (44).

##### 2.2.3.3. Hamburg Quality of Life Questionnaire in Multiple Sclerosis

The Hamburg Quality of Life Questionnaire in Multiple Sclerosis (HAQUAMS) is a MS-specific, validated and reliable instrument measuring QoL. Version 10.0 of the HAQUAMS consists of 44 items, mostly using a 5-point Likert scale. Twenty eight of these items are subdivided into six subscales for “upper extremity,” “lower extremity,” “cognition,” “fatigue,” “mood,” and “communication.” Remaining items cover additional symptom domains as well as a patient-rated estimation of disease progression over time and main impairments. Mean subscale scores are calculated, HAQUAMS total score is computed by the means of the six subscale scores with lower scores corresponding to a higher quality of life (45).

##### 2.2.3.4. Physical function

The 9-Hole Peg Test (9-HPT) assesses the manual dexterity and is a quick motor function test of the upper extremity to determine fine motor skills. It is a valid test used routinely in clinical practice and research. Results of measured manual dexterity are reliable to distinguish between pwMS and healthy subjects. The test is performed twice with the dominant hand and twice with the non-dominant hand. The mean value from the four measurements is subsequently calculated and given as mean seconds (46). With the Timed 25-Foot Walk (T25-FW), a walking distance of 25 feet (7.62 meters) is measured in seconds. The T25-FW is known to be a highly reliable and valid tool to capture change in ambulatory disability and is established in clinical settings (47).

### 2.3. Data analyses

#### 2.3.1. Descriptive analyses

Descriptive analyses were performed for all of the collected and measured data to gain knowledge about the current health behaviors in this clinical pwMS cohort. Categorical variables were summarized as absolute and relative frequencies, and continuous variables were summarized as mean and standard deviation (SD). These results were compared to other published MS cohorts and the general German population using data from the “German Health Update” conducted by the Robert Koch Institute (RKI) in the German adult population (2014/15 and 2020/21) (48).

#### 2.3.2. Regression analyses

Based on current literature regarding health behaviors in pwMS and MS-related disease outcomes we hypothesized that diet, physical activity, tobacco smoking and BMI might be associated with cardiovascular comorbidities, QoL and MS-specific physical impairment. We chose BMI as an independent variable instead of waist circumference as recent studies using this measure showed associations with MS disease outcomes (21, 49). Models were adjusted for sex, age, disease duration and school education as covariates based on clinical and previous research experience. Beyond this, no further variable selection was undertaken. Regression analyses were carried out to explore possible associations using SPSS Version 27. Self-reported diet score (sDQS, continuous), tobacco smoking status (categorial), the physical activity score (GLTEQ, continuous) as well as BMI (continuous) were used as independent variables. No further variable selection was done. Multiple regression analyses were performed with the number of complete cases for each of the three outcome variables (a) physical impairment (T25-FW, 9-HPT), (b) QoL (HAQUAMS total score and subscores mood, fatigue, cognition) and (c) cardiovascular comorbidities. EDSS was not used as an outcome variable for physical impairment as the intervals between values are not equal. This property disqualified the EDSS for inclusion in a linear regression analysis. The first models (a) physical impairment and (b) quality of life were run as linear regression analyses. In addition a binary logistic regression analysis was performed to explore the associations with (c) cardiovascular comorbidities (0 = no comorbidities, 1 = one or more comorbidities). The significance level was set at 5% (two-sided).

#### 2.3.3. Sample size calculation

With a sample size of 381, a small to moderate effect size of *f*^2^ = f 0.035 was considered to be detectable by testing five independent variables and controlling for another six variables. The power was set as 80% and the type I error at 5% (two-sided hypothesis). *f*^2^ is defined as ρ^2^/(1-ρ^2^) where ρ^2^ is the squared multiple correlation coefficient of the variables being tested. The sample size calculation was conducted with PASS 15.

## 3. Results

### 3.1. Descriptive analyses

From August 2020 to June 2021 *n* = 894 pwMS were treated in the MS Outpatient Clinic of the UKE. Main characteristics such as age, disease duration and disability in the studied cohort (*n* = 399) did not meaningfully differ from the patient cohort (*n* = 495) presenting at the clinic, but not included in the study during the same time period (see Table 1). We defined a complete case cohort providing data in all questionnaires with *n* = 134 cases. All 3 samples were compared. Results showed that the main characteristics such as age, disease duration and disability of complete cases were similar to the studied cohort with some bias of complete cases in favor of younger female pwMS with higher education and less physical impairment (data not shown).

**Table 1:** Comparison of the convenience sample with patients from the MS Day Clinic who were not included in the study during the same period (August 2020–June 2021).

	Convenience sample *N* = 399	Clinical cohort not included in study *N* = 495	*p*
**Socio-demographic characteristics**			
Age in years, mean (SD)	41.8 (12.8)	42.1 (12.8)	0.70^a^
Missing, *n*	1	0	
Sex, *n* (%)			0.55^b^
Males	135 (34)	177 (36)	
Females	263 (66)	317 (64)	
Missing, *n*	1	1	0.83^c^
School education, *n* (%)			0.21^b^
<12 years	192 (49)	112 (54)	
>12 years	200 (51)	94 (46)	
Missing, n*	7	289	<0.01^c^
**MS-specific data**			
EDSS, mean (SD)	2.77 (1.9)	2.56 (2.0)	0.12^a^
Missing, *n*	22	7	

MS: Multiple sclerosis, SD: Standard deviation, EDSS: Expanded Disability Status Scale.

a *t*-test.

b Chi-squared test.

c Chi-squared test with missing as a separate category.

*Education in the clinical cohort (*n* = 495) was often not recorded due to a communication error.

Table 2 summarizes the characteristics of the study cohort. Mean age was 42 years (SD 12.8) with a mean disease duration since diagnosis of 7.4 years (SD 8.4). 66% were female and the most frequent disease course was RRMS (52.1%). Physical impairment was low as measured by a mean EDSS of 2.8 (SD 1.9). 50% of pwMS in this study sample were overweight including 19% being obese. The mean BMI was 25.9. A high waist circumference was measured in 53% of female and 40% of male pwMS in this study sample. Mean sDQS score was 38.8 which is indicative of a moderately healthy diet. Daily fruit and vegetable consumption was reported by 63.8% of all participants. This was reported in 65.4% of the female population and 60.7% of the male population. Physical activity measured with the GLTEQ HCS showed that 36.8% could be classified as physically active persons and 19.8% as moderately active. Almost half (43.5%) were characterized as insufficiently active. Looking at the smoking status, 24.4% reported to be current tobacco smokers vs. 75.7% current non-smokers [never-smoker (43.9%), ex-smoker (31.8%)]. The highest percentage of current smokers was in males between 18 and 29 years (47.4%). 27.3% of the sample reported one or more cardiovascular comorbidities.

**Table 2:** Sample description with socio-demographic characteristics, behavioral characteristics, cardiovascular risk factors, cardiovascular comorbidities and MS-specific data.

Variables	Convenience sample *N* = 399	N missing
**Socio-demographic characteristics**		
Age in years, mean (SD)	41.8 (12.8)	1
**Sex**		1
Males, *n* (%)	135 (34)	
Females, *n* (%)	236 (66)	
**Behavioral characteristics**		
**Diet (sDQS)**		113
sDQS score, mean (SD)	38.82 (4.3)	
Insufficiently healthy, *n* (%)	2 (1)	
Moderately healthy, *n* (%)	154 (54)	
Healthy, *n* (%)	130 (46)	
**Fruit and vegetable intake**		1
Daily fruit and vegetable consumption, *n* (%)	254 (64)	
Males, *n* (%)	82 (61)	
Females, *n* (%)	172 (65)	
**Physical activity (GLTEQ)**		70
Health contribution score, mean (SD)	20.07 (21.1)	
Insufficiently active (%)	143 (44)	
Moderately active (%)	65 (20)	
Active (%)	121 (37)	
**Tobacco smoking**		9
Smoker, *n* (%)	95 (24)	
Non-smoker, *n* (%)	171 (44)	
Ex-smoker, *n* (%)	124 (32)	
**Cardiovascular risk factors**		
**Blood pressure**		11
Systolic blood pressure, mean (SD)	132.48 (19.1)	
Diastolic blood pressure, mean (SD)	81.74 (11.4)	
Measured hypertension, *n* (%)	80 (20.6)	
**Body Mass Index (BMI)**		9
BMI, mean (SD)	25.88 (5.38)	
Underweight, *n* (%)	10 (3)	
Normal weight, *n* (%)	186 (48)	
Pre-obesity, *n* (%)	122 (31)	
Obesity, *n* (%)	72 (19)	
**Waist circumference**		5
Waist circumference females, mean (SD)	83.66 (16.04)	
High waist circumference females ≥80 cm, *n* (%)	138 (53)	
Waist circumference males, mean (SD)	90.72 (15.15)	
High waist circumference males ≥94 cm, *n* (%)	54 (40)	
**Self-reported cardiovascular comorbidities**		
Hyperlipedemia, *n* (%)	37 (9)	21
Hypertension, *n* (%)	70 (18)	21
Diabetes type 1, *n* (%)	9 (2)	35
Diabetes type 2, *n* (%)	10 (3)	23
Peripheral vascular disease, *n* (%)	2 (1)	26
Heart disease, *n* (%)	15 (4)	25
No comorbidities, *n* (%)	290 (73)	0
One or more comorbidities, *n* (%)	109 (27)	0
**MS-specific data**		
Duration of disease, mean in years (SD)	7.38 (8.44)	44
Disease course		59
SPMS, *n* (%)	44 (13)	
RRMS, *n* (%)	177 (52)	
PPMS, *n* (%)	55 (16)	
MS type not yet defined, *n* (%)	56 (17)	
Other, *n* (%)	8 (2)	
Expanded Disability Status Scale, mean (SD)	2.77 (1.94)	22
Timed 25 foot walk, mean in sec. (SD)	5.58 (2.5)	26
9 Hole Peg Test, mean in sec. (SD)	24.15 (8.86)	9
**Quality of life (HAQUAMS)**		
Total score, mean (SD)	2.22 (0.77)	99
Subscore mood, mean (SD)	2.54 (0.92)	33
Subscore fatigue, mean (SD)	2.59 (1.18)	48
Subscore cognition, mean (SD)	2.36 (1.13)	55

SD, Standard deviation. MS: Multiple sclerosis, sDQS: short Diet Quality Screener, GLTEQ: Godin Leisure Time Exercise Questionnaire, SPMS: Secondary progressive MS, RRMS: Relapsing-remitting MS, PPMS: Primary progressive MS.

### 3.2. Associations with physical impairment

The multiple linear regression model testing the association between independent variables and 9-HPT included 206 complete records (Table 3, corrected *R*^2^ = 0.18). The four variables sDQS, school education, sex and disease duration were significantly associated with the result of the 9-HPT. With a healthier diet the upper limb impairment was lower (*b* = −0.30, 95% CI [−0.58, −0.02], *p* = 0.033) and higher in persons who are longer affected by MS (*b* = 0.42, 95% CI [0.27, 0.57], *p* < 0.001) and attended school for less than 12 years (*b* = 2.46, 95% CI [0.17, 4.75], *p* = 0.035). The model showed that females performed better on 9-HPT than men (*b* = −3.60, 95% CI [−6.04, −1.16], *p* = 0.004). The multiple linear regression model testing for T25-FW included 197 complete records (Table 4, corrected *R*^2^ = 0.184). Analyses showed that T25-FW performance was worse in pwMS with lower education (<12 years, *b* = 0.82, 95% CI [0.07, 1.56], *p* = 0.032) and with higher age (*b* = 0.07, 95% CI [0.04, 0.11], *p* < 0.001).

**Table 3:** Results multiple linear regression analysis for manual dexterity (9-Hole-Peg Test), *n* = 206.

	*b*	95% CI		*p*
		Lower	Upper	
Constant term	37.9	25.28	50.52	<0.001
**Variables**				
sDQS	−0.30	−0.58	−0.02	0.033
Non-smoker vs. ex-smoker	−0.46	−3.14	2.22	0.733
Smoker vs. ex-smoker	−2.86	−6.18	0.45	0.090
GLTEQ	−0.01	−0.06	0.04	0.658
BMI	−0.12	−0.32	0.07	0.216

Corrected *R*^2^ = 0.18.

sDQS: short Diet Quality Screener, GLTEQ: Godin Leisure Time Exercise Questionnaire, BMI: Body Mass Index, b: unstandardized regression coefficient, CI: confidence interval.

Adjusted for age, sex, duration of disease and school education.

**Table 4:** Results multiple linear regression analysis for mobility (Timed 25-Foot Walk Test), *n* = 197.

	*b*	95% CI		*p*
		Lower	Upper	
Constant term	1.16	−2.94	5.26	0.577
**Variables**				
sDQS	−0.01	−0.97	0.83	0.875
Non-smoker vs. ex-smoker	0.67	−0.21	1.255	0.134
Smoker vs. ex-smoker	−0.01	−1.08	1.06	0.986
GLTEQ	0.002	−0.01	0.02	0.785
BMI	0.02	−0.05	0.08	0.629

Corrected *R*^2^ = 0.14.

sDQS, short Diet Quality Screener; GLTEQ, Godin Leisure Time Exercise Questionnaire; BMI: Body Mass Index.

b: unstandardized regression coefficient, CI: confidence interval.

Adjusted for age, sex, duration of disease and school education.

### 3.3. Association with quality of life

The multiple linear regression model testing the association between independent variables and the HAQUAMS total score included 163 complete records (Table 5, corrected *R*^2^ = 0.09). Multiple linear regression analyses were performed for the HAQUAMS total score and for the subscores mood, fatigue and cognition. The results of the subscores are not shown in the table due to the similarity to the results of the total score. With longer disease duration, quality of life (HAQUAMS total score) was lower (*b* = 0.03, 95% CI [0.01, 0.04], *p* < 0.001).

**Table 5:** Results multiple linear regression analysis for self-reported MS-specific quality of life (HAQUAMS total score), *n* = 163.

	*b*	95% CI		*p*
		Lower	Upper	
Constant term	2.19	0.76	3.620	0.003
**Variables**				
sDQS	−0.02	−0.05	0.02	0.342
Non-smoker vs. ex-smoker	0.09	−0.20	0.37	0.556
Smoker vs. ex-smoker	0.11	−0.24	0.47	0.531
GLTEQ	−0.001	−0.01	0.01	0.846
BMI	−0.001	−0.02	0.02	0.919

Corrected *R*^2^ = 0.09.

sDQS, short Diet Quality Screener; GLTEQ, Godin Leisure Time Exercise Questionnaire; BMI, Body Mass Index, b: unstandardized regression coefficient, CI: confidence interval.

Adjusted for age, sex, duration of disease and school education.

### 3.4. Association with cardiovascular comorbidities

The binomial logistic regression included 207 complete records testing the association between independent variables and cardiovascular comorbidities (Table 6). Of the eight variables entered into the regression model, BMI and age were significantly associated with having one or more comorbidities, while all other variables (sDQS, smoking status and GLTEQ) showed no significant association. The chance of having one or more comorbidities increases by 17%, if the BMI increases by one point (OR = 1.17, 95% CI [1.10, 1.26], *p* < 0.001) and increases by 8% with each additional year of life (OR = 1.08, 95% CI [1.04, 1.12], *p* < 0.001).

**Table 6:** Results binary logistic regression analysis for self-reported cardiovascular comorbidities, *n* = 207.

	OR	95% CI		*p*
		Lower	Upper	
**Variables**				
sDQS	1.04	0.95	1.15	0.397
Smoking status				0.196
Non-smoker vs. smoker	1.00	0.36	2.76	0.999
Ex-smoker vs. smoker	0.45	0.14	1.40	0.169
GLTEQ	1.00	0.99	1.02	0.690
BMI	1.17	1.10	1.26	<0.001

Cox & Snell *R*^2^ = 0.20, Nagelkerkes *R*^2^ = 0.30.

sDQS, short Diet Quality Screener; GLTEQ, Godin Leisure Time Exercise Questionnaire; BMI, Body Mass Index, OR: odds ratio, CI: confidence interval.

Adjusted for age, sex, duration of disease and school education.

## 4. Discussion

To the best of our knowledge, this is the first study describing various health behaviors in a German clinical cohort of pwMS and at the same time exploring possible associations with physical impairment, quality of life and cardiovascular comorbidities.

### 4.1. Modifiable risk factors

#### 4.1.1. Diet

The mean sDQS score of 38.8 in this clinical cohort, which is indicative of a moderately healthy diet, is comparable with two studies among pwMS [AMSLS and Health Outcomes and Lifestyle Intervention in a Sample of pwMS (HOLISM)] which also used a dietary screener, the Dietary Habits Questionnaire (DHQ, range 20–100), to assess the health value of participants’ dietary patterns. Participants from the AMSLS cohort (*n* = 1,490) (12) achieved a mean score of 73.4, whereas participants of the HOLISM study (*n* = 2087) scored 79.0 (11). Participants of the HOLISM study have been recruited through interactive online sites specifically for pwMS from 52 countries including the USA, Australia, UK, New Zealand and Canada and were asked to fill out an online survey. Although these dietary screeners are not fully comparable, we note that in all study samples participants reached about 2/3 of the achievable points and thus a moderate diet quality can be assumed. Looking at fruit and vegetable consumption as an indicator of a healthy dietary pattern, a higher percentage of pwMS in this clinical cohort reported consuming fruits and vegetables daily (females 65.4%, males 60.7%) compared to participants of the Gesundheit in Deutschland aktuell (GEDA) 19/20 cohort (females 45.1%, males 24.1%) (50).

#### 4.1.2. Tobacco smoking

Reports from the webbased NARCOMS registry (11.1%) (16) show a substantially lower proportion of active smokers compared to this German clinical MS cohort (24%) and to the German National MS (NationMS) cohort (31.6%) (21). This is of concern, because smoking rates in Germany are known to be substantially higher compared to other Western or North European countries (51). Representative data consistently show tobacco smoking rates of 28% in the adult general German population (50, 51). Smoking cessation seems to significantly reduce disability progression in pwMS (52, 53). Therefore it would be recommendable that clinicians inform pwMS, who are persistent smokers, with evidence-based information about the impact of smoking cessation directly after diagnosis and at the same time provide assistance for those who are willing to quit smoking.

#### 4.1.3. Physical activity

A distribution of physical activity levels similar to the results of this study was observed in our earlier webbased study among pwMS (*n* = 1.027 from all over Germany) on long-term adherence to exercise, 45.3% were active (54). Comparison with the GEDA 2019/20 study is difficult because investigators used a questionnaire based on WHO recommended guidelines on Physical Activity for Health which differs from the criteria of the GLTEQ. Therefore, a comparison to the general German population is not possible. A meta-analysis showed that pwMS are less physically active than a general population (55).

#### 4.1.4. Body Mass Index

BMI results in this clinical cohort (mean BMI = 25.8) are in line with representative data of the German population from the GEDA Cohort 2019/20 (50) (Mean BMI = 26.4) and data from the GEDA Cohort 2014/15 (56) which showed that 18.1% of participants were obese. Moreover, our data are also similar to those of a large international sample (HOLISM) of *n* = 2.469 pwMS with prevalence rates of 23.3% for overweight and 18.6% for obesity (11). Data from the NARCOMS registry with a size of *n* = 5.832 from the US showed a mean BMI of 27 (57) which is slightly more as measured in our cohort. A substantial difference is the mean age of 60 years of the NARCOMS population vs. a mean age of 42 years in our cohort. A comparable sample of *n* = 470 pwMS with a mean age of 37.5 years was recruited from the Kaiser Permanente Southern California (KPSC) database and showed similar prevalence rates of 31.1% for overweight, but a higher rate of 36.4% for obesity (33). As BMI tends to increase with progressing age, it is concerning that this German, relatively young sample is already overweight on average. Comparison with the German NationMS cohort—a more active and younger (median age 31 y) sample of *n* = 1,066 recruited in MS centers early in the disease—show a lower median BMI of 24.2 and a lower rate of 15% for obesity (21).

### 4.2. Associations

#### 4.2.1. Physical impairment

Exploring possible associations between health behaviors and physical impairment, no significant associations were found except for diet quality. A higher sDQS score was associated with less impairment of manual dexterity as measured with the 9-HPT, but not with ambulatory disability as measured with the T25-FW. We assume that this rather is a chance finding, because otherwise we would have expected an association with ambulatory disability as well. Moreover, other studies also found contradictory results: The Ausimmune study (12) also did not find an association between total diet score and levels of disability as measured with the Patient Determined Disease Steps (PDDS) scale, whereas results from the NARCOMS registry (8) and the HOLISM study (11) indicate lower levels of disability for participants with higher diet quality scores. These heterogeneous study results might be explained by the use of different outcome measures, dietary assessment tools, recruitment strategies (registry data vs. online survey vs. clinical sample) and hence participant samples with differing mean age and disease duration. Moreover, findings from cross-sectional studies are prone to different kinds of biases, causal inferences cannot be drawn and results need to be verified in longitudinal and at best interventional studies which are scarce. To date, systematic reviews have shown that consistent evidence for dietary interventions in persons with MS is lacking and the evidence base is weak (58, 59).

We did not find significant associations between other health behaviors and physical impairment, which is somewhat surprising, because other studies reported less disability with increasing physical activity (60, 61) and more disability (62) as well as faster progression to SPMS (52) among pwMS who were smoking (63). There are several possible explanations for the divergent results in this study compared to other studies. First, participants in our cohort were younger (mean age 42 years), had a shorter disease duration (mean 7 years) and accordingly lower disability (mean EDSS 2.8) compared to most other cohorts (8, 12, 23, 26, 63). It can be assumed that the negative effects of poor health behaviors only become apparent with older age and longer disease duration.

#### 4.2.2. Quality of life

We did not find any significant associations between health behaviors or BMI with QoL in this study. This is in contradiction to results from the Australian AMSLS study (*n* = 1,490) by Marck et al. (12), who found that a higher total diet score was associated in a dose–response manner with better physical and mental quality of life. However, in line with the results of this study Marck et al. (12) did not find associations of total diet score with level of mobility limitations, fatigue and other common MS symptoms influencing MS-related QoL. In comparison to association studies using diet scores, Coe et al. (9) investigated the influence on MS-specific outcomes of specific foods/nutrients in a large cohort of the UK Multiple Sclerosis Registry (*n* = 2,410). They found that a high fiber and fruits/vegetable consumption was associated with a better health-related QoL, whereas the consumption of red meat showed inverse results (9). A recent systematic review and meta-analysis on the impact of dietary interventions on fatigue and quality of life among pwMS found 12 trials comparing eight dietary interventions. While some diets (e.g., Mediterranean, Paleolithic) showed reductions in fatigue and improvements in quality of life compared to control, the overall credibility of evidence was graded very low due to high or moderate risk of bias, small sample sizes, and the limited number of studies included in the network meta-analysis (64).

While previous analyses have shown mixed evidence regarding the effect of exercise and physical activity on QoL (18, 65), a recent systematic review and meta-analysis found moderate positive effects (66). Registry data from the NARCOMS indicate, that active smoking was associated with significantly lower health-related QoL on most subscales (16). Our study could not confirm these findings, possibly due to the low disability level of this clinical cohort.

#### 4.2.3. Cardiovascular comorbidities

Meta-analytic estimates from population-based studies among persons with MS revealed prevalence rates of 18.6% for hypertension, 10.9% for hyperlipidemia and 8.6% for type 2 diabetes (67), which aligns well with the results of our study (hypertension 17.5%, hyperlipidemia 9.3%). Only type 2 diabetes (2.5%) was reported less frequently in our clinical MS cohort which might be explained by the relatively young age of our sample. Compared to recent data from the German MS registry where 17.3% of pwMS had cardiovascular comorbidities, in our sample approximately 10% more pwMS reported one or more cardiovascular comorbidities (27%) (68). Moreover, males and females in our clinical cohort show higher rates of self-reported hypertension (18.8% of females, 18.3% of males) than the self-reported frequency of hypertension in the general German population in the age group of 30–44 (9% of females, 14.5% of males) (69). This is of concern, because prevalence of comorbidities increases with age, and it can be assumed that elderly pwMS will additionally suffer from age-related comorbidities (70). As especially comorbidities, which are known to be influenced by health behaviors such as physical activity and diet are associated with a higher disease activity and progression (25), pwMS should be informed and encouraged to prevent comorbidities by modifying their health behaviors.

We did not find significant associations between smoking behavior, diet or physical activity and the presence of self-reported cardiovascular comorbidities. However, every one-point-increase in BMI was associated with a 17% increased chance of suffering from cardiovascular comorbidities. A scoping review including 17 observational and 17 interventional studies indicates evidence that a higher level of physical activity could improve vascular comorbidity risk in pwMS. As a higher BMI is linked to reduced physical activity and poor diet quality, we would therefore also have expected associations between these health behaviors and cardiovascular comorbidities in this study (71). Several studies provide strong evidence that the presence of comorbidities is associated with more brain atrophy (72), higher lesion burden (73) and a worse MS prognosis (23, 26). Therefore, comorbidity management should be suggested in overall MS care (25). Moreover, analyzing data from nearly 3,000 pwMS from the Veterans Affairs MS National Data Repository, Turner et al. (63) found that the presence of comorbidities was associated with a 10% increased risk of mortality over a 15-year period. In addition, baseline tobacco smoking was associated with a significantly higher and physical activity with a significantly lower mortality risk. A possible influence of diet quality was not examined in this study.

### 4.3. Strengths and limitations

The present study has two considerable strengths. First, we were able to show that the examined convenience sample did not differ significantly from the total cohort, which was examined in the MS Outpatient Clinic in the same time period. The sample examined can therefore be considered representative of pwMS, who turn to a university-based outpatient clinic. Moreover, the sample showed suboptimal health behaviors similar to the German general population. The second strength is the inclusion of all major health behaviors/related factors (dietary behavior, physical activity, smoking and BMI) in all multivariable regression models, which are assumed to function as independent variables in each model. We aimed to avoid that independent variables not included in the model compensate a causal effect by indirect correlations. In comparison to some other association studies, this study included all major potentially influencing variables at the same time. The ambitious aim led, among other factors as explained in detail in the limitations section, to the small number of included pwMS in each regression analysis.

Our study also had some limitations. First, recruitment in a clinical sample is prone to missing data, which was aggravated by difficulties in clinical routine due to the Covid-19 pandemic. Secondly, missing data in the regression analyses has arisen due to the strict data completeness criterium explained in the previous paragraph, we applied for a case to be included: for conducting a regression analysis not just each question for the outcome variable (e.g., cardiovascular comorbidities with six questions) but also all 22 questions/measurements representing the independent variables (sDQS, Smoking, GLTEQ and BMI) and the four variables (age, sex, school education and disease duration) for adjustment had to be completed. The high number of questions that needed to be completed, apparently led to the limited case number. This resulted in lower numbers of participants that could be included in the regression analyses, which resulted in reduced statistical power. Hence, we cannot completely rule out that issues of participation bias have contributed to the lack of associations between health behaviors and outcomes in this study. Although we could show that the studied convenience sample was representative for pwMS presenting at a large university-based outpatient clinic did not meaningfully differ from the whole patient cohort treated in the MS Outpatient Clinic during the same time period, it cannot be assumed that the sample examined is representative of the general MS population in Germany and results consequently can only be partially transferred.

Moreover, we did not validate self-reported comorbidities with clinical records or insurance company data and relied on self-report data for health behaviors. Although validated tools were used, findings might be confounded by treated comorbidities, which pwMS might not have reported as they are controlled under treatment. This could have affected the accuracy of data. A general issue in this field is the use of different scales and measurement tools when assessing health behaviors and outcomes, e.g., health related QoL. A defined set of scales used across all MS studies in different settings and countries is lacking. This might be an explanation for differing results of our study compared to other studies. Finally, causal inferences cannot be drawn due to the cross-sectional design of the study.

## 5. Conclusion

This relatively young clinical cohort of pwMS shows a high prevalence of critical health behaviors and cardiovascular comorbidities, which is of concern. While associations between modifiable risk factors and physical impairment, QoL and cardiovascular comorbidities were small or not significantly evident, we believe that the relatively short disease duration and relatively young age substantially contributed to this result. The negative effects of poor health behaviors might only become apparent with older age and longer disease duration. Hence, from a clinical point of view, it still seems warranted to further investigate associations of health behaviors with MS outcomes in clinical populations. Accordingly, we believe that there is an urgent need for targeted interventions to improve health behaviors among pwMS, especially smoking cessation programs and interventions that aim at regaining or maintaining normal body weight. In addition to DMTs, pwMS should be motivated and supported to implement long-term health behavior changes at an early stage of disease such as a healthy diet, regular physical activity and smoking cessation.

## Data availability statement

The raw data supporting the conclusions of this article will be made available by the authors, without undue reservation.

## Ethics statement

This study involving humans was carried out in accordance with the recommendations of Ethics Committee of Ärztekammer Hamburg. The protocol was approved by the Ethics Committee of Ärztekammer Hamburg (PV 4405). The studies were conducted in accordance with the local legislation and institutional requirements. The participants provided their written informed consent to participate in this study.

## Author contributions

KG: writing—original draft and investigation. CH, KG, and KR-L: conceptualization. AD, CH, HS, JP, KG, KR-L, and NK: methodology. AD and KG: formal analysis. JP and KG: data curation. CH and KR-L: supervision, project administration, and funding acquisition. AD, CH, HS, JP, KR-L, and NK: writing—reviewing and editing. All authors contributed to the article and approved the submitted version.

## Funding

This work was funded by the Deutsche Forschungsgemeinschaft (DFG, German Research Foundation) project no. -495901503. The funders had no role in study design, data collection and analysis, decision to publish, or preparation of the manuscript.

## Conflict of interest

The authors declare that the research was conducted in the absence of any commercial or financial relationships that could be construed as a potential conflict of interest.

## Publisher’s note

All claims expressed in this article are solely those of the authors and do not necessarily represent those of their affiliated organizations, or those of the publisher, the editors and the reviewers. Any product that may be evaluated in this article, or claim that may be made by its manufacturer, is not guaranteed or endorsed by the publisher.

## Appendix

**Table A1:** Scoring method for the Diet Quality Index.

1. Daily frequency consumption during the last 4 weeks Please think about the last 4 weeks. How often per day did you eat the following foods in the specified amounts?					
Food	Amount	<1 time/day	1 time/day	≥2 times/day	Never
Whole grain bread	1–2 slices	1	2	3	1
Vegetables/salad	1 serving	1	2	3	1
Fruits	1 piece or serving	1	2	3	1
Yoghurt/milk/cheese	1 tub/1 glass/1 slice	1	2	3	1
Whole grain products (e.g., Pasta, rice)	1 serving	1	2	3	1
Plant oil (e.g., olive or sunflower)	1 tablespoon	1	2	3	1
Calorie free drinks (e.g., water, tea)	3 glasses of 250 mL	1	2	3	1
**2. Weekly frequency consumption during the last 4 weeks** **Please think about the last 4 weeks. How often per week did you eat the following foods in the specified amounts?**					
**Food**	**Amount**	**<4 times/week**	**4–6 times/week**	**≥7 times/week**	**Never**
Meat	1 serving ~150 g	3	2	1	3
Cold cuts, sausages/ meat products (e.g., meatball)	1–3 slices, 1 serving	3	2	1	3
Pastry or cake	1–2 pieces	3	2	1	3
Sweets	1 serving	3	2	1	3
Butter or lard	1 teaspoon	3	2	1	3
Sweetened beverages (e.g., Cola)	1 glass	3	2	1	3
Fast food	1 serving	3	2	1	3
**Food**	**Amount**	**<1 time/week**	**1 time/week**	**≥2 times/week**	**Never**
Fish	1 serving	1	2	3	1
Legumes	1 serving	1	2	3	1
**Food**	**Amount**	**<2 times/week**	**2–3 times/week**	**≥4 times/week**	**Never**
Unsalted nuts, oilseeds	1 handful	1	2	3	1
